# Effect of thermal and non-thermal processing on fermentable oligo-di-monosaccharides and polyols (FODMAPs) content in millet, sorghum, soybean and sesame varieties

**DOI:** 10.3389/fnut.2025.1520510

**Published:** 2025-03-11

**Authors:** Docus Alowo, Solomon Olum, Ivan Muzira Mukisa, Duncan Ongeng

**Affiliations:** ^1^Department of Food Science and Postharvest Technology, Faculty of Agriculture and Environment, Gulu University, Gulu, Uganda; ^2^Department of Food Innovation and Nutrition, Faculty of Agriculture and Environmental Sciences, Mountains of the Moon University, Fort Portal, Uganda; ^3^Department of Food Technology & Nutrition, School of Food Technology, Nutrition & Bioengineering, College of Agricultural and Environmental Sciences, Makerere University, Kampala, Uganda

**Keywords:** fermentable oligo-di-monosaccharides and polyols, grain variety, roasting, malting, cereals, legumes, fructo-oligosaccharides, galacto-oligosaccharides

## Abstract

This study investigated the effect of processing (roasting and malting) and crop variety on fermentable oligo-di-monosaccharides and polyols (FODMAPs) profile of millet, sorghum, soybean, and sesame varieties commonly consumed in Uganda. Two elite varieties and one indigenous variety for each crop were analyzed. Monosaccharide and polyols content was determined by HPLC-UV method, while disaccharides and oligosaccharide were determined using Megazyme kits. The elite varieties of soybean (Maksoy 3 N), Millet (Seremi 2) and sorghum (Narosorg 2) exhibited significantly (*p* < 0.05) lower oligosaccharide content compared to indigenous varieties with percentage differences ranging from 10.2 to 73.9%. Additionally, Maksoy 3 N and Narosorg 2 also exhibited significantly lower (*p* < 0.05) excess fructose content compared to the indigenous variety. Malting was more effective than roasting (*p* < 0.05) in reducing FODMAP categories and total FODMAP content. Malting effectively reduced excess fructose in all grain types to the recommended levels of <0.15 g/100 g compared to roasting. Moreover, malting reduced total oligosaccharides and total FODMAPs in soybean and sesame by more than 50%. However, this reduction did not achieve the recommended threshold of 0.3 g/100 g for total oligosaccharides and 0.5 g/100 g, for total FODMAPs which are a criterion to categorize low FODMAP diets. Malting conditions should be optimized to enhance its effectiveness in producing low FODMAP foods. This study highlights the importance of selecting appropriate grain variety and processing techniques that modify FODMAP content in foods that can be used for dietary therapy of gastro-intestinal disorders among vulnerable population.

## 1 Introduction

Cereal grains, oil seeds and legumes such as wheat, rice, millet, sorghum, sesame, and soybean constitute significant sources of nutritionally valuable compounds globally. These compounds include protein, carbohydrates, fiber, vitamins, minerals, and functional bioactive compounds such as polyphenols, carotenoids, and oligosaccharides (1). The unique balance of bioactive components and prebiotics (Fructo-oligosaccharides (FOS) and galacto-oligosaccharides (GOS)) in these grains have attracted substantial attention in the field of food and nutritional sciences. Bioactive compounds in plant-based foods have been associated with positive nutritional and health outcomes such as amelioration of non-communicable diseases (2) and functional gastro-intestinal disorders (FGID) (3). Consequently, research focus in food and nutritional sciences has shifted from basic nutrition to exploring the health-promoting properties of foods. Understanding how these compounds contribute to overall health is crucial for developing effective dietary interventions especially in this era of personalized nutrition.

More than 70% of households in sub-Saharan Africa, including Uganda, rely on these plant-based foods as significant sources of nutrients and bioactive compounds (1). To address the demand for plant-based foods and adapt to climate change, crop breeding technologies have been adopted to develop crop varieties that are high yielding and resilient to various environmental stressors (4). In Uganda, these elite varieties have predominantly replaced the indigenous crop varieties in diets and food processing industries. Elite crop varieties of sorghum (SESO and Narosorg series), soybean (Namsoy and Maksoy series), sesame (Sesim series), and millet (Seremi and Naromil series) have been highly adopted and consumed by Ugandan households (4, 5). These varieties are preferred for their superior traits, including high yield, good fermentation and nutritional qualities, and increased resistance to pests, diseases and drought (4, 6). They are widely utilized in both households and food industries as ingredients to produce a range of products including complementary foods, local dishes, non-alcoholic and alcoholic beverages (7, 8).

The health benefits associated with plant-based foods are strongly influenced by the composition and structure of various bioactive compounds such as prebiotics and polyphenols. For instance, cereal grains, and legumes contribute to improved gastro-intestinal health due to their substantial amounts of prebiotic carbohydrates (9). These carbohydrates, also known as fermentable oligo-di-monosaccharides and polyols (FODMAPs) (10), undergo fermentation, resulting in a reduced pH and increased concentration of short-chain fatty acids (11). These effects promote health by creating an unfavorable environment for pathogenic microorganisms and supporting the growth of beneficial gut microflora (12). FODMAPs that are selectively utilized by host microorganisms, promoting their growth and producing health-beneficial metabolites, are generally classified as prebiotics (13).

However, not all FODMAPs possess prebiotic properties, as FODMAPs represent a broader category of fermentable carbohydrates, some of which can have adverse effects on the host. For instance, foods with high levels of fructose and polyols can lead to flatulence, bloating and abdominal distention (14) due to gas production and increased water volume by osmosis in the lower gastro intestinal tract (15). Additionally, these substrates are associated with gut microbiota dysbiosis (16). Therefore, characterization and quantification of FODMAP content in commonly consumed plant-based foods becomes inevitable if effective targeted dietary therapy is to be achieved.

The FODMAP profiles of cereals, specifically wheat, rye, and barley, as well as legumes such as chickpeas, green peas, faba beans, soybean, red kidney beans and lentils, have been studied. These studies report FOS as the predominant FODMAP in the bran of cereals (17) while the legumes are rich in raffinose family GOS (18). However, there is a lack of information on the FODMAP profiles of other commonly consumed cereals in sub-Saharan Africa such as millet and sorghum. Similarly, there is limited data on the FODMAP profiles of oil seeds such as sesame, pumpkin seeds, and sunflower seeds despite their frequent use in plant-based diets of various households. Furthermore, several studies have investigated the effects of food processing on cereals, legumes and oil seeds. These studies have examined the impact of processing on functional properties (19), proximate and micronutrient composition (20), and sensory attributes (21). Nevertheless, there are few limited studies (22–25) focused on processing techniques specifically aimed at altering FODMAP content in cereals, legumes and oil seeds to meet the recommended reference cut of values for a low FODMAP diet (26), which is commonly used to alleviate FGID. None of the above authors focused on millet, sorghum, sesame and soybean varieties despite their significant inclusion in sub-Saharan African diets.

Studies on how processing techniques and grain variety influence FODMAP content of these crop varieties are limited. Existing information is derived from studies that focus on processing effects related to nutritional and physicochemical properties (59, 60), whereas varietal effects are predominantly studied in terms of yield stability and adaptability to varying environmental conditions (4, 61). Diverse processing methods, including thermal techniques such as dry heating and baking, and nonthermal techniques such as malting, fermentation, and enzymatic hydrolysis, significantly alter the functional properties of FODMAPs (25, 62). These techniques primarily alter the degree of polymerization and hence the structural composition of the grains. The extent of alteration of FODMAP content depends on the inherent structural composition, type, initial quantity of FODMAP and grain variety (27). However, the extent to which these processing methods and grain variety affect individual FODMAPs is not known.

Additionally it is important to note that the quality and quantity of FODMAPs in legumes, oil seeds and cereal grains are influenced by various environmental conditions and varieties across the globe (26). Thus, extrapolating data from one continent to another may result in erroneous estimates, either overestimating or underestimating the FODMAP content. Consequently, this renders dietary interventions for specific nutritional diseases, such as FGIDs, more speculative than effective. Moreover, it makes it challenging to achieve effective personalized nutrition. This study, therefore investigated the effect of thermal and non-thermal processing and crop variety on the FODMAP profile of millet, sorghum, soybean, and sesame varieties commonly consumed in Uganda.

## 2 Materials and methods

Sugar standards (monosaccharide kit 47,267, sugar alcohol kit 47,266) and other chemicals (acetonitrile, methanol, sodium borohydride, *p*-aminobenzoic ethyl ester ABEE-derivatizing agent, HPLC-grade chloroform) were purchased from Sigma Aldrich, United States. Analytical grade glacial acetic acid was sourced from Merck (Millipore, Darmstadt, Germany). HPLC grade water and ultra-pure water were obtained from water purification system (Evoqua, Ultrapure water system Art. No. W3T364778, Germany). All chemicals and reagents were stored following the manufacturer’s instructions.

### 2.1 Selection of samples

This study focused on four crops, including two cereals: finger millet (*Eleusine coracana*) and sorghum (*Sorghum bicolor*), one legume: soybean (*Glycine max*) and one oil seed: sesame (*Sesamum indicum*). These grains are recognized sources of FODMAPs with prebiotic potential including FOS (fructo-oligosaccharides) and GOS (galacto-oligosaccharides) (3). Each grain type comprised of two elite varieties and one indigenous variety commonly consumed by Ugandan Households. The indigenous grains were collected from five different grocery and produce stores across various markets in Gulu City, northern Uganda. Equal quantities of each grain were combined to make 2 kg samples for each type. The selected elite varieties originated from two national plant breeding institutes: the National Semi-Arid Resources Research Institute (NASARRI), Serere district, Uganda, and the Ngetta Zonal Agricultural Research and Development Institute, Lira district, Uganda. The criteria for variety selection included their suitability for both food consumption and brewing as previously reported by Byakika et al. (28).

### 2.2 Sample treatment

Individual grains were thoroughly sorted and cleaned with portable water. The samples were subsequently oven-dried at 50°C for 24 h to a consistent weight. Approximately 500 g of each dried grain was subjected to either thermal processing (dry heat-roasting) or non-thermal processing (malting), while the unprocessed grains served as control. For thermal processing, the method described by Kim et al. (29) was followed with minor adjustments in roasting temperatures and holding time. Specifically, the grains were roasted on an electric hot plate (DLAB MS-H280-Pro, Los Angeles United States) at temperatures ranging from 90°C to 100°C, with a holding time of 10 min for sorghum, millet, and sesame, and 20 min for soybean grains. These conditions are considered adequate to significantly reduce the beany flavor and antinutritional factors in the grains while preserving nutritional quality (30).

Regarding malting, the method described by Syeunda et al. (31) was employed. Briefly, cleaned grains were steeped in distilled water (500 g of grain to 1.5 L of water) for 24 h. The steeping water was changed every 6 h to ensure uniform air circulation, and the final water was drained prior to sprouting. Subsequently, they were allowed to sprout at an ambient temperature (25–27°C) for 48 h. During the sprouting stage, the grains were uniformly mixed and periodically moistened to facilitate homogenous sprouting and temperature regulation. After 48 h, the sprouted grains were kilned in an oven at 50°C until a moisture content of 10% was achieved. Finally, the raw, roasted, or malted grains were each ground using a laboratory blender (Neo-Tech SA, Milmort, Belgium) and passed through a 300 μm screen. The flour samples were sealed in high-density polyethylene bags and stored at 8°C prior to subsequent analysis.

### 2.3 Carbohydrate extraction

The water and ethanol extraction method was used as described by Schmidt & Sciurba (32) with minor modifications. Briefly, 2.0 g of each sample was mixed with 4 mL of 80% ethanol (v/v) to inactivate amylase and prevent excessive starch hydrolysis during the extraction process (33). Exactly 20 mL of distilled water was added followed by sonication (GT SONIC-D9, Beijing, China) at room temperature for 3 min. Sonication was conducted to enhance percentage yield during the extraction process (34). The mixture was then centrifuged (VWR Micro star 17, 521–1,646, Berlin, Germany) at 3000 x g for 5 min and the resulting supernatant was collected. FODMAPs were further re-extracted from the residue with 20 mL of distilled water. The supernatants for the two extractions were pooled and made up to 100 mL. Each sample underwent three independent extractions, with each extraction replicated twice during analysis to account for variability in the extraction and analysis process.

### 2.4 HPLC determination of monosaccharide and polyol content in the samples

#### 2.4.1 Preparation of standard and sample solution

Standards (Glucose, fructose, sorbitol and mannitol) and sample solutions were prepared according to the method of Jalaludin and Kim (35).The sample extracts were derivatized with *p*-aminobenzoic acid ethyl ester (ABEE) according to the method described by Debebe et al. (36). Briefly, ABEE stock solution was prepared by dissolving 2 g of ABEE agent in 4 mL of methanol in a 1 L glass tube followed by addition of 310 mL of glacial acetic acid. The mixture was completely dissolved at 45°C in a water bath and left to cool to room temperature. Upon cooling, 0.6 g of sodium borohydride were added, and the solution was vortexed for 30 s to yield an ABEE stock solution.

A 2 mL aliquot of the ABEE stock solution was mixed with 500 μL of either standard or sample solution and incubated at 80°C for 1 h. The mixture was cooled under running water and centrifuged (Eppendorf AG Barkhausenweg 122,339 Hamburg, Germany) at 4000 x g for 1 min. Exactly 3 mL of HPLC grade water was added to the supernatant followed by addition of 3 mL of chloroform to extract the aqueous phase. The mixture was vortexed for 30 s and again centrifuged at 4000 x g for 1 min. The upper layer was collected, centrifuged (4,000 x g for 1 min), and filtered into HPLC vials using a 0.22 μm nylon syringe filter (AVF-100C-NY, Dubai, UAE) for HPLC analysis.

#### 2.4.2 HPLC analysis

HPLC was performed following the procedure of Debebe et al. (36) with a slight modification in conditions. The analysis was performed using a C18 column (Luna^R^ 5 μm C18 100 Å, LC Column 250 × 4.6 mm) and UV detector at λ_max_ 190 nm. This wavelength yielded a consistent correlation coefficient of 0.99 for both monosaccharides and polyols indicating good detector response. Two mobile phases (75% Acetonitrile as solvent A and HPLC water as solvent B) were used in a gradient elution mode. The gradient conditions were 0–4 min with 65% solvent A, which linearly increased to 80% within 5–10 min followed by maintenance at 80% for 11–12 min with a mobile-phase flow rate of 0.5 mL·min^−1^. The sample injection volume was 10 μL with the column temperature set at 40°C.

The data was evaluated using a calibration method where standards (Glucose, fructose, sorbitol, and mannitol) of 6 different concentrations ranging between 0.039–10 μg/mL were used to create calibration curves. A correlation coefficient of >0.98 was accepted. The calibration curve was used to determine the limit of quantification (LOQ) of the standards using Equation 1. The precision of the analysis was checked after 4 repeated injections of each standard. The set of the standards was run at the start and at the end of the sample running session to allow correction of any drift in the elution profile. Quantitative determination of the individual monosaccharide was by external standard method. The summary of LOQ ranges, correlation coefficients and R^2^ values for each sugar standard are indicated in Table 1. Chromatograms obtained were analyzed using LabSolutions CS software version 5 (Shimadzu LC-2050, Tokyo Japan) and the concentration of the corresponding sugar was calculated by obtaining the area under the curve.


1 $$LOQ=10\;x\;\left(\mathrm{standard}\;\mathrm{deviation}\;\mathrm{of}\;\mathrm{intercept}÷\mathrm{slope}\right)$$

**Table 1:** The correlation coefficient, R^2^ and limit of quantification ranges of sugar standards.

Compound	Correlation coefficient R	R^2^	Equation	LOQ (μg/ml)
Fructose	0.999	0.999	y = 13,226x-3433.5	1.77–10
Glucose	0.998	0.997	y = 21,586x + 1813.5	3.276–10
Sorbitol	0.998	0.997	y = 22,780x + 1728.9	3.56–10
Mannitol	0.996	0.993	y = 22,202x + 9256.8	5.1–10

### 2.5 Determination of disaccharide and oligosaccharide contents

Disaccharide and oligosaccharide content in the samples were prepared using Megazyme kits and determined by UV–VIS spectrophotometer (Jenway 6,705, Milmort Belgium). K-RAFGL kit (last updated August 2023) was used to determine sucrose, and galacto-oligosaccharide content whereas total fructo-oligosaccharide was determined by K-FRUC kit (last updated November 2022; Megazyme, Bray Ireland). During determination of Fructo-oligosaccharides, *α*-galactosidases (Megazyme cat. no. EAGLANP) was used to eliminate interfering GOS. The kits were used following manufacturer’s instructions. A calibration curve was created for 6 different concentrations of each standard and LOQ for the compounds was determined using equation 1. The LOQ ranges of FOS and GOS were between 27.1–250 μg/mL and 123.3–250 μg/mL and the correlations coefficients (R) were 0.998 and 0.995, respectively. The R^2^ values and equations were 0.997, y = 0.0099x + 0.0222 and 0.991, y = 0.0044x + 0.0479, for FOS and GOS, respectively.

### 2.6 Determination of total FODMAP content in millet, sorghum, soybean and sesame

The total FODMAP content in the different grains and seeds was calculated according to Varney (26). The individual contents of monosaccharide, disaccharides, oligosaccharides, and polyols in each sample were summed to calculate the total FODMAP content.

### 2.7 Statistical analysis

Data for FODMAP content was analyzed using one-way Analysis of Variance (ANOVA). Mean comparisons were performed using the Tukey’s HSD test and *α* = 0.05 was used to detect significant differences in means. Prior to performing ANOVA, the test for homogeneity of variance was confirmed using Levene’s test and normal distribution was ascertained using Shapiro–Wilk test. All statistical analyses were performed using SPSS software version 25.0 (SPSS Inc., Chicago, IL). Finally, graphical illustrations were generated using Sigma Plot version 11.0.

## 3 Results

### 3.1 Characterization of fermentable oligo-di-monosaccharides and polyols in selected grains and seeds

Table 2 illustrates variations in FODMAPs content according to seed type. Generally, the FODMAPs profiles were influenced by crop type. Glucose was significantly higher (*p* < 0.05) in millet compared to other crops. Fructose on the other hand was not significantly different from the crops except in soybean that registered a significantly lower (*p* < 0.05) fructose content by 0.26–0.29 g/100 g. For disaccharides, sucrose content in soybean was about 3 times higher than in sesame, sorghum and millet. Similarly, soybean had the highest (*p* < 0.05) GOS and FOS content followed by sesame. Sugar polyols were below the limit of quantification values for all grains and seeds under study.

**Table 2:** Characterization of FODMAP profiles of millet, sorghum, soybean and sesame.

Crop type	Monosaccharides/disaccharides (g/100 g)				Polyols (g/100 g)	Oligosaccharides (g/100 g)	
	Glucose	Fructose	Excess fructose	Sucrose	Sorbitol/mannitol	Total GOS	Total FOS
Soybean	0.05^b^ ± 0.03	0.08^b^ ± 0.04	0.03^b^ ± 0.06	3.37^a^ ± 0.4	< LOQ	4.43^a^ ± 0.85	3.7^a^ ± 0.43
Millet	0.28^a^ ± 0.15	0.37^a^ ± 0.05	0.09^ab^ ± 0.19	0.51^c^ ± 0.17	< LOQ	< LOQ	0.57^b^ ± 0.33
Sorghum	0.14^b^ ± 0.03	0.34^a^ ± 0.05	0.2^a^ ± 0.08	0.41^c^ ± 0.07	< LOQ	0.22^c^ ± 0.1	0.63^b^ ± 0.48
Sesame	0.07^b^ ± 0.04	0.35^a^ ± 0.09	0.28^a^ ± 0.11	0.97^b^ ± 0.09	< LOQ	1.93^b^ ± 0.43	0.83^b^ ± 0.23

Values show mean ± SD (*n* = 6) for each crop type; values in the same column with different superscript are significantly different (*p* < 0.05); Excess Fructose, Fructose-Glucose; LOQ, Limit of Quantification (sorbitol LOQ = 3.56 μg/mL, mannitol LOQ = 5.1 μg/mL & total GOS LOQ = 27.1 μg/mL).

### 3.2 Effect of crop variety on the contents of fermentable oligo-di-monosaccharides and polyols

Significant differences (*p* < 0.05) in FODMAP content were observed between elite and indigenous varieties, with varying magnitudes (Table 3). The elite soybean (Maksoy 3 N) and sorghum varieties, (Narosorg 2) exhibited significantly lower (*p* < 0.05) excess fructose content compared to the indigenous variety. The excess fructose content in Narosorg 2 was significantly lower by 0.12 and 0.17 g/100 g compared to the indigenous and Narosorg 4 varieties, respectively.

**Table 3:** Influence of crop variety on fermentable oligo-di-monosaccharides and polyols content in Millet, Sorghum, Soybean and Sesame.

		**Monosaccharides (g/100g)**			**Disaccharides (g/100g)**	**Oligosaccharides (g/100g)**	
**Crop**	**Variety**	**Glucose**	**Fructose**	**Excess fructose**	**Sucrose**	**Total GOS**	**Total FOS**
Soybean	Maksoy 3N	0.06^a^±0.01	0.03^b^±0.01	-	3.78^a^±0.05	4.56^a^±0.09	3.52^a^±0.03
	Maksoy 6N	0.06^a^±0.02	0.09^b^±0.04	0.03^a^±0.02	3.31^b^±0.28	3.48^b^±0.16	3.80^a^±0.32
	Indigenous variety-soybean	0.08^a^±0.10	0.12^a^±0.01	0.04^a^±.01	3.03^b^±0.07	5.13^a^±0.44	3.90^a^±0.58
Millet	Naromil 2	0.14^c^±0.03	0.40^a^±0.01	0.26^a^±0.02	0.7^a^±0.05	0.03±0.17	0.81^a^±0.19
	Seremi 2	0.27^b^±0.01	0.39^a^±0.01	0.12^a^±0.01	0.49^b^±0.01	< LOQ	0.4^b^±0.19
	Indigenous variety-millet	0.47^a^±0.07	0.31^a^±0.06	-	0.32^c^±0.09	< LOQ	0.58^b^±0.31
Sorghum	Narosorg 2	0.18^a^±0.01	0.26^b^±0.05	0.09^b^±0.05	0.31^b^±0.02	0.18^b^±0.02	0.52^b^±0.08
	Narosorg 4	0.12^b^±0.02	0.38^a^±0.01	0.26^a^±0.01	0.45^a^±0.05	0.17^b^±0.08	0.21^b^±0.03
	Indigenous variety-sorghum	0.13^b^±0.01	0.34^a^±0.04	0.21^a^±0.04	0.48^a^±0.02	0.32^a^±0.06	1.13^a^±0.29
Sesame	Sesim 2	0.10^a^±0.04	0.25^a^±0.14	0.16^a^±0.18	1.00^a^±0.08	2.30^a^±0.25	0.86^a^±0.07
	Sesim 3	0.06^a^±0.04	0.32^a^±0.10	0.26^a^±0.11	0.9^a^±0.05	1.86^ab^±0.04	0.73^a^±0.19
	Indigenous variety-sesame	0.05^a^±0.01	0.33^a^±0.1	0.27^a^±0.08	0.98^a^±0.07	1.5^b^±0.24	1.0^a^±0.37

Values show mean ± SD (n=6) for each grain variety; values in the same column with different superscript are significantly different (*P* < 0.05); Excess Fructose=Fructose-Glucose; -; no excess fructose available; LOQ; Limit of Quantification (Total GOS = 27.1 μg/mL).

Similarly, the elite varieties of soybean, sorghum and millet demonstrated significantly lower (*p* < 0.05) total oligosaccharide content except in Naromil 2 millet variety which exhibited significantly higher (*p* < 0.05) values. Specifically, the indigenous variety of sorghum had significantly higher (*p* < 0.05) GOS (by 56%), and FOS (by 73.9%) compared to the elite varieties. Additionally, the percentage difference in GOS and FOS content between the indigenous soybean variety and the elite varieties ranged from 10.2 to 38.3%. Conversely, oligosaccharide content in sesame elite and indigenous varieties were not significantly different (*p* > 0.05). By implication therefore, varietal effect due to crop improvement technologies such as conventional breeding had a non-uniform effect on FODMAP content within crop types.

### 3.3 Effect of processing on the contents of fermentable oligo-di-monosaccharides and polyols

Table 4 shows the effect of processing techniques on FODMAP contents in oil seeds, cereals, and legume grains investigated. The results indicate that the type of processing significantly (*p* < 0.05) influenced FODMAP quantities, regardless of grain type albeit at varying magnitude. Malting demonstrated a greater potential to reduce FODMAPs in millet, sorghum, soybean and sesame compared to roasting. Malting significantly (*p* < 0.05) increased glucose levels across all grains, with the highest increase observed in sorghum (428.6%) and millet (250%). Conversely, malting significantly (*p* < 0.05) reduced fructose levels in millet, sorghum, and sesame, with sorghum showing the greatest reduction (79.4%), while soybean exhibited an increase of over 100%. Additionally, malting significantly (*p* < 0.05) reduced galacto-oligosaccharides (GOS), especially in soybean (71.6%) and sesame (62.2%). However, no significant reduction in GOS was observed in sorghum and millet. The effect of malting on fructo-oligosaccharides (FOS) was variable, increasing in cereals (11.1% in sorghum, 63.2% in millet) but significantly reducing in sesame and soybean by 14.5 and 68.9%, respectively.

**Table 4:** Effect of processing on FODMAPs content in millet, sorghum, soybean and sesame.

Crop	Type of processing	Monosaccharides/disaccharides (g/100 g)			Oligosaccharides (g/100 g)		Total FODMAP
		Glucose	Fructose	Excess fructose	Total GOS	Total FOS	(g/100 g)
Soybean	Unprocessed	0.05^b^ ± 0.03	0.08^c^ ± 0.04	0.03^c^ ± 0.06	4.43^a^ ± 0.85	3.70^a^ ± 0.43	8.16^a^ ± 0.85
	Roasting	0.07^ab^ ± 0.04	0.49^a^ ± 0.03	0.41^a^ ± 0.05	4.34^a^ ± 0.95	3.59^a^ ± 0.41	8.34^a^ ± 1.28
	Malting	0.14^a^ ± 0.06	0.28^b^ ± 0.03	0.15^b^ ± 0.05	1.26^b^ ± 0.90	1.15^b^ ± 0.73	2.56^b^ ± 2.95
Millet	Unprocessed	0.28^b^ ± 0.15	0.37^ab^ ± 0.05	0.09^a^ ± 0.19	< LOQ	0.57^a^ ± 0.33	0.65^a^ ± 0.41
	Roasting	0.3^b^ ± 0.14	0.45^a^ ± 0.1	0.17^a^ ± 0.15	< LOQ	0.48^a^ ± 0.44	0.65^a^ ± 0.37
	Malting	0.98^a^ ± 0.33	0.27^b^ ± 0.15	–	< LOQ	0.93^a^ ± 0.38	0.93^a^ ± 0.38
Sorghum	Unprocessed	0.14^b^ ± 0.14	0.34^b^ ± 0.05	0.2^a^ ± 0.08	0.22^a^ ± 0.10	0.63^a^ ± 0.48	1.05^a^ ± 0.58
	Roasting	0.17^b^ ± 0.17	0.42^a^ ± 0.04	0.25^a^ ± 0.09	0.30^a^ ± 0.14	0.36^a^ ± 0.13	0.91^a^ ± 0.25
	Malting	0.74^a^ ± 0.74	0.07^c^ ± 0.03	–	0.20^a^ ± 0.15	0.70^a^ ± 0.86	0.90^a^ ± 0.89
Sesame	Unprocessed	0.07^b^ ± 0.04	0.35^ab^ ± 0.09	0.28^a^ ± 0.11	1.93^a^ ± 0.43	0.83^a^ ± 0.23	3.04^a^ ± 0.36
	Roasting	0.04^b^ ± 0.02	0.45^a^ ± 0.09	0.41^a^ ± 0.10	2.46^a^ ± 0.41	0.49^a^ ± 0.28	3.36^a^ ± 0.64
	Malting	0.18^a^ ± 0.03	0.26^b^ ± 0.07	0.08^b^ ± 0.08	0.73^b^ ± 0.65	0.71^a^ ± 0.54	1.52^b^ ± 0.65

Values show mean ± SD (*n* = 6) for each grain type. Means in the same column with different superscripts are significantly different (*p* < 0.05). Excess Fructose, Fructose-Glucose. –, no excess fructose available. Total FODMAP = (Excess Fructose + total GOS + total FOS); LOQ, LOQ-Limit of Quantification (total GOS LOQ = 27.1 μg/mL).

In contrast, roasting did not significantly affect GOS and FOS irrespective of the crop type. However, it resulted in a significant (*p* < 0.05) increase in fructose levels across all grains, ranging from 21.6 to 512.5%. Soybean and sesame showed the highest increase.

### 3.4 The potential of roasting and malting in achieving low FODMAP food products

**Figure 1:**
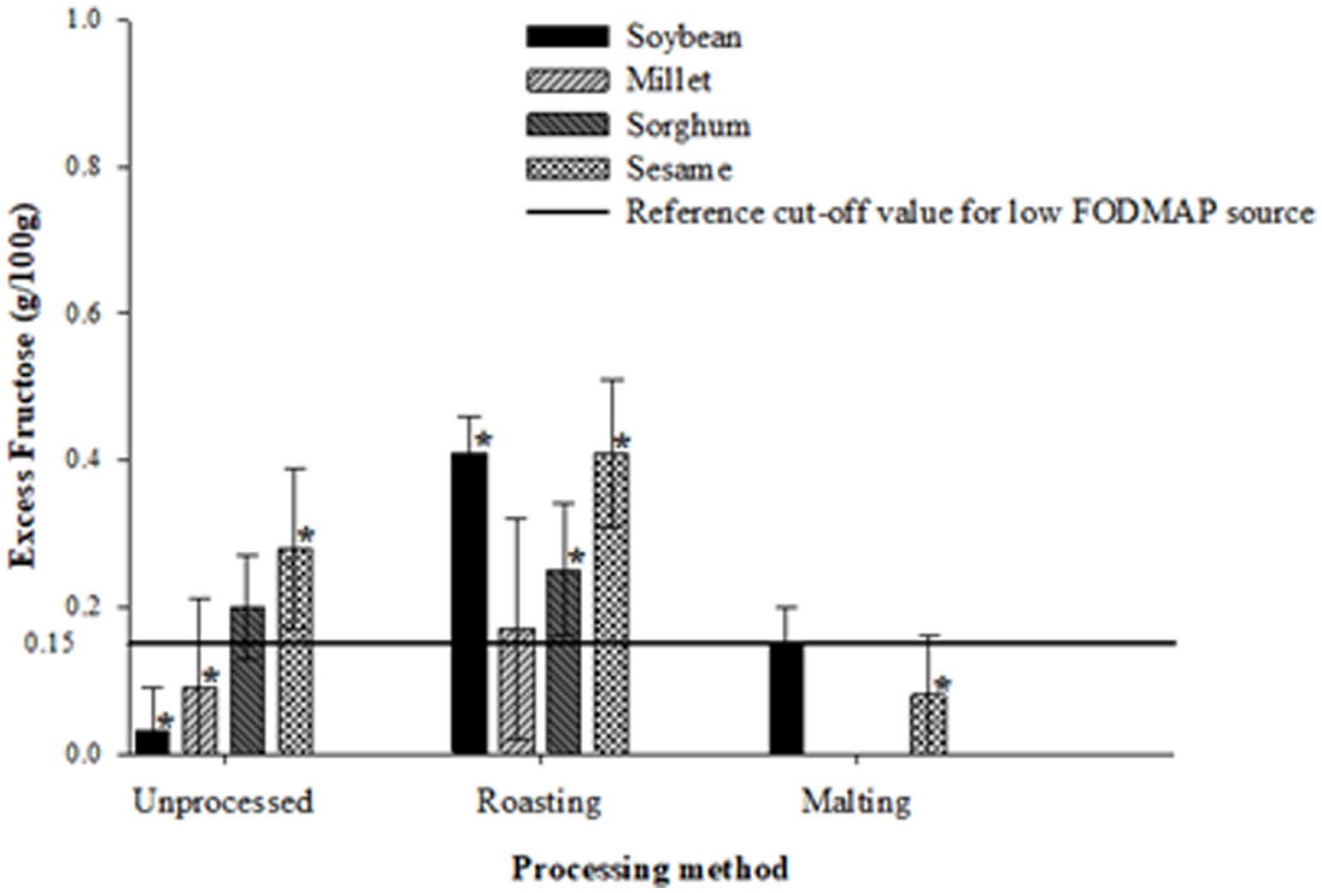
Effect of processing on excess fructose content ratings of millet, sorghum, soybean and sesame compared to the reference cut-off value for a low FODMAP food (26). Values are means of three independent determinations. Error bars represent standard deviation. Graphs with asterisk are significantly different (*p* < 0.05) from the reference cut-off value.

**Figure 2:**
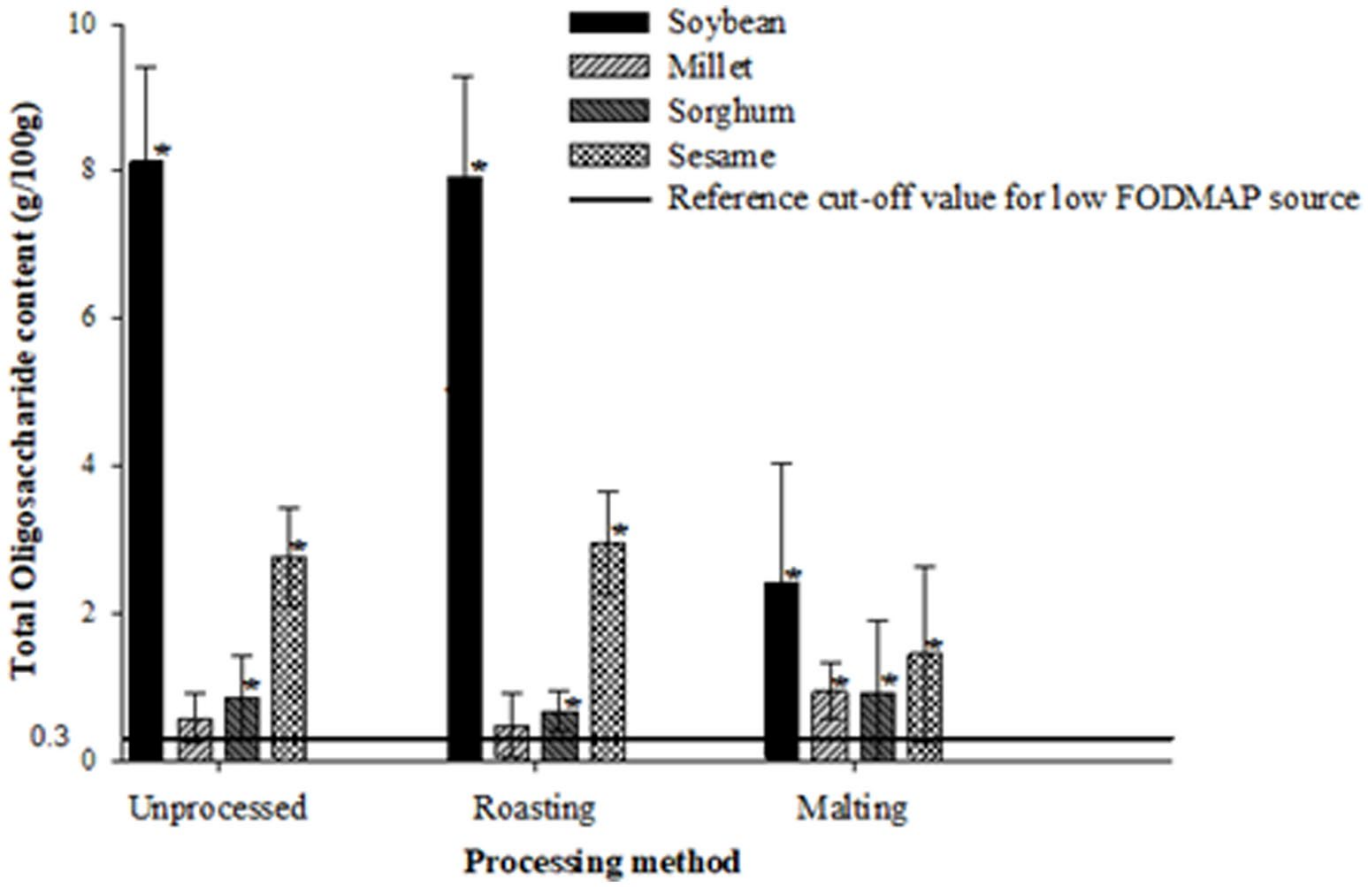
Effect of processing on total oligosaccharide content ratings of millet, sorghum, soybean and sesame compared to the reference cut-off value for a low FODMAP food (26). Values are means of three independent determinations. Error bars represent standard deviation Graphs with asterisk are significantly different (*p* < 0.05) from the reference cut-off value.

**Figure 3:**
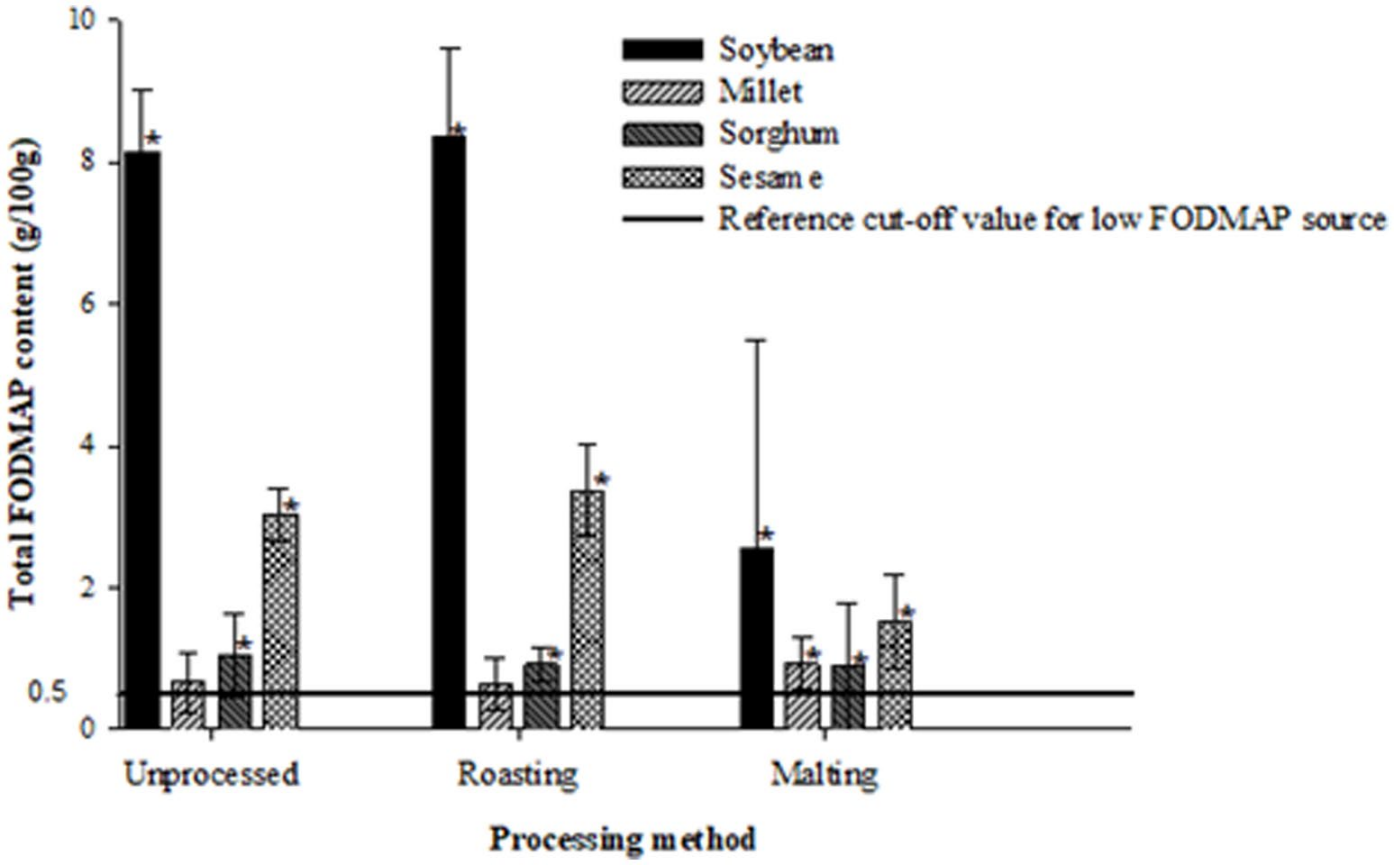
Effect of processing on total FODMAP content ratings of millet, sorghum, soybean and sesame compared to the reference cut-off value for a low FODMAP food (26). Values are means of three independent determinations. Error bars represent standard deviation. Graphs with asterisk are significantly different (*p* < 0.05) from the reference cut-off value.

Figures 1–3 illustrate the potential of roasting and malting in reducing FODMAPs to the recommended threshold values for each FODMAP category with the objective of obtaining a low FODMAP food product. Malting effectively reduced excess fructose in all grain types to the recommended levels of <0.15 g/100 g, a reduction which was not achieved by roasting (Figure 1). The amount of excess fructose obtained after malting was not significantly different (*p* > 0.05) from the reference cut-off value for soybean but was significantly lower (*p* < 0.05) in sesame, millet and sorghum. After malting, millet and sorghum did not exhibit any excess fructose content. Furthermore, malting had a greater effect on reducing the total oligosaccharides and total FODMAPs in soybean and sesame (>50% reduction) although this reduction did not result in attaining the recommended threshold of 0.3 g/100 g (Figure 2) and 0.5 g/100 g (Figure 3), respectively. Specifically, the reduction of both total oligosaccharides and total FODMAP remained significantly higher (*p* < 0.05) in all crop types than the reference cut-off values.

Conversely, roasting significantly increased (*p* < 0.05) excess fructose, total oligosaccharide, and total FODMAP content in soybean, sesame, and sorghum compared to the reference cut-off values. Millet, however, did not show any significant difference (*p* > 0.05) from all the reference cut-off values. The percentage differences ranged from 50 to 185.4%, with sorghum exhibiting the lowest and soybean the highest significant differences from the reference cut-off values.

## 4 Discussion

### 4.1 Characterization of fermentable oligo-di-monosaccharides and polyols in selected grains and seeds

Understanding the characteristics of fermentable oligo-di-monosaccharides in cereal, legume and oil seeds provides an insight to allow for: (i) selection of nutritious and functional crop type for use in dietary modification therapies (22), (ii) enhancing the content of bioactive compounds through food processing (37), and (iii) updating the food composition tables (38). The characteristics of FODMAPs reported in this study delineated sesame, soybean, sorghum and millet as crops with varying quantities of FODMAPs. They can be used as good candidates for modification through processing to develop products with lower FODMAP content as dietary interventions for FGIDs. This information can also be used to update information in the current food composition tables for East and central Africa because these crop varieties are consumed in the two African regions (39).

The results obtained in the current study are comparable with those of Ispiryan et al. (18) who reported legume such as peas and beans to contain substantial quantities of galacto-oligosaccharide (between 4.48 to 4.87 g/100 g) and total oligosaccharide. Galacto-oligosaccharides and fructo-oligosaccharides are known prebiotic substrates that modulate gut microbiota and inhibit pathogen growth (40). Conversely, the higher content of fructose in sorghum, millet and sesame may contribute to fructose-related gastrointestinal digestive disorders such as increased motility and flatulence in sensitive individuals. This is further emphasized by Larke et al. (41) who reported increased gastrointestinal inflammation among adults who frequently consumed fructose rich diets.

These findings highlight the dual role of FODMAP components in gastrointestinal health emphasizing the necessity for personalized dietary recommendations tailored to individual needs. For instance, individuals predisposed to gastrointestinal disorders may benefit from a diet rich in Oligosaccharides such as those in soybean (Maksoy 3 N). Conversely, individuals sensitive to monosaccharides should limit their intake of foods like sorghum and sesame. This personalized approach could enhance overall gut health and reduce the risk of digestive disorders contributing to the achievement of Sustainable Development Goal 3, which aims at achieving good health and well-being.

### 4.2 Effect of crop variety on the contents of fermentable oligo-di-monosaccharides and polyols

The observed significant variation in FODMAP content between elite and indigenous varieties of millet, sorghum, soybean and sesame highlight the impact of crop improvement technologies such as conventional breeding on carbohydrate composition in cereals, legumes and oil seeds. These variations were comparable to those reported by Bhardwaj (42) in S26 and S32 sesame varieties and Karnpanit et al. (27) in Australian sweet Lupin legumes. Moreover, Raja et al. (43) reported variations in GOS content in different chickpea varieties while Huynh et al. (44) reported variations in FOS content in different wheat and barley varieties.

These varietal differences in FODMAP content could be attributed to crop improvement technologies that aim at improving agronomic characteristics potentially at the expense of total nutritional content and quality. Furthermore genotypic differences and varying geographical environmental conditions (26) could also be a plausible explanation for the differences in FODMAP content. The varying FODMAP content reported in elite varieties has three major implications in the field of nutrition. Firstly, these varieties may increase the symptoms of functional gastrointestinal disorders in sensitive individuals especially those with high fructose (45). Secondly, those with higher total oligosaccharides (FOS & GOS) can provide ameliorating effect to FGIDs among vulnerable population such as children under 5 years of age. This is due to their proven prebiotic properties (46). Thirdly, the high FODMAP content presents an opportunity to apply food processing technologies to develop symbiotic food products. Symbiotic food products contain both prebiotic and probiotic properties (47). Therefore, careful selection of crop varieties is essential to balance between nutritional benefits with potential digestive health implication.

### 4.3 Effect of processing on the contents of fermentable oligo-di-monosaccharides and polyols

The results of this study, which demonstrated a reduction of more than 50% in FODMAP content due to malting, are consistent with results from previous studies. Nyyssölä et al. (24) reported a 90% decrease in galacto-oligosaccharide (GOS) content in faba beans and yellow peas following enzymatic treatment. Similarly, Kaczmarska et al. (48) reported a 44% reduction in total oligosaccharides in germinated soybean whereas Ispiryan et al. (23) documented a 60–85% decrease in GOS content in malted chickpeas, barley, and oats. During malting, GOS are rapidly mobilized by endogenous catabolic *α*-galactosidases during the germination stage (49). This endogenous enzyme activation is crucial for energy metabolism to promote plant growth during the initial stages (50). This could be a plausible explanation for the reduction of GOS in malted plant-based foods such as soybean, sesame, millet, and sorghum.

The varying effect of malting on FOS content may be attributed to differences in grain type, inherent FOS quantity and type, and processing steps. Cereals such as wheat, rye and barley, millet and sorghum may contain varying quantities of levan and inulin type fructo-oligosaccharides with varying glycosidic linkages of either β-2 → 1 or β-2 → 6 fructose-sucrose glycosidic linkages (51). These linkages influence the degree of polymerization which in turn influences their ability to be broken down by malting. However, this study did not explore this perspective in detail.

Conversely, the effect of roasting was more visible at the total FODMAP level rather than individual FODMAP categories. This could be attributed to re-polymerization and re-construction of new carbohydrate glycosidic linkages (52) and moisture removal during roasting leading to increased concentration of FODMAPs (53). Overall, the study demonstrates that processing techniques significantly alter FODMAP content depending on the FODMAP category and processing method. Malting is more effective than roasting in reducing FODMAP content in cereals and legumes, making it a preferable method for producing low-FODMAP foods. Malting is effective in reducing FODMAP categories such as excess fructose with adverse effects upon consumption by vulnerable population such as children under 5 years.

In most Ugandan households, millet, sesame, sorghum, and soybean are fundamental in the diet and nutrition. Millet and sorghum serve as staple cereals, frequently used to prepare porridge, non-alcoholic fermented beverages, bread, and other traditional dishes (7). Similarly, soybean and sesame are utilized either as single ingredients or in combination to develop snacks, composite flours, and pastes, contributing to the diversity and nutritional value of the diet (20, 54). Malting these food ingredients can lead to production of various food products with low FODMAP content while preserving nutritional quality.

### 4.4 The potential of roasting and malting in achieving low FODMAP food products

Functional gastro-intestinal disorders (FGIDs) incidences are increasing in developing countries, and dietary management plays a crucial role in symptom control. Despite high FODMAP foods being known triggers for FGID symptoms (45), they are also rich in nutrients. Therefore, elimination or significant reduction of daily intake of these foods may lead to nutrient deficiencies (55). Previous studies have documented that bioprocessing methods such as malting and fermentation can reduce FODMAP content while maintaining nutritional quality (25). These methods also produce symbiotic food products with prebiotic and probiotic properties (56).

The results of this study indicate that malting significantly reduces FODMAP contents in cereal and legume grains commonly consumed in Uganda and other countries in eastern and central Africa. However, reductions attained could not achieve the recommended cut-off values of below 0.3 g/100 g and 0.5 g/100 g for total oligosaccharides and total FODMAP, respectively (26). Similar results were reported by Ispiryan et al. (23), for chickpea (0.49 g/100 g) and lentil malt (0.84 g/100 g). Schmidt & Sciurba (32) also found comparable FODMAP content in fermented wheat flour varieties (0.53 g/100 g). The failure to attain cut-off values as observed in the current study could be due to the initially high FODMAP content in cereals and legumes investigated and ineffectiveness of selected processing conditions. Therefore, optimization of processing parameters for the methods investigated could be explored to improve their effectiveness in producing low FODMAP food products. Optimizing these processes is crucial for developing dietary interventions that minimize gastrointestinal discomfort for individuals sensitive to FODMAPs, particularly in regions where these grains are staple foods.

Based on the results of this study and others (55, 63), it is plausible to suggest that the 0.5 g/100 g threshold for a low FODMAP diet (26), may be excessively restrictive for high FODMAP food resources such as cereals, legume grains, and oil seeds used in this current study. Given that FODMAPs confer health benefits primarily through prebiotic mechanism of action (57), reducing them to such low levels might undermine these benefits. Notably, the FODMAP levels achieved in this study through malting fall within the recommended prebiotic intake range of 5–8 g/day, which is believed to be adequate to elicit a prebiotic effect in the host (58). However, the current study did not explore this aspect. Therefore, future studies should explore the prebiotic potential of FODMAPs at levels achieved through malting for the promising varieties of millet, soybean, sorghum and sesame identified in this study. This will provide a basis for striking a compromise between achieving the recommended FODMAPs threshold and the expected prebiotic benefit. These quantities maybe protective from functional gastrointestinal disorders rather than act as potential triggers.

## 5 Conclusion

This study successfully characterized the FODMAP content in cereals, oil seeds, and legumes varieties commonly consumed in Uganda and demonstrated the potential of malting to reduce FODMAP content to recommended cut-off values. Specifically, the study demonstrated that; (i) Crop improvement leads to varying changes in the total FODMAP content of elite crop varieties. The elite varieties of soybean (Maksoy 3 N), millet (Seremi 2) and sorghum (Narosorg 2) contain lower total FODMAP content compared to indigenous varieties. Therefore, careful selection of crop varieties is essential to balance between nutritional benefits with potential digestive health implication; (ii) malting is more effective than roasting at reducing total FODMAP content, especially fructose which is known to have adverse digestive health effects when consumed in high quantities. Despite its superiority at reducing FODMAPs compared to roasting, malting conditions still need to be optimized to be able to achieve the recommended threshold of 0.5 g/100 g recommended for low FODMAP diets.

## Data Availability

The original contributions presented in the study are included in the article/supplementary material, further inquiries can be directed to the corresponding author.
